# Migraine as an occupational health risk among nurses in Qassim region, Saudi Arabia: a multicenter study of risk perceptions and associated factors

**DOI:** 10.3389/fpubh.2026.1789630

**Published:** 2026-05-14

**Authors:** Shereen Ahmed A. Qalawa, Mohamed Goda Elbqry, Fatma Mohamed Elmansy, Samia Eaid Elgazzar, Fatima S O Ashmieg, Saddam Ahmed Al-Ahdal, Aljoud Abdulrahman Alshitwy

**Affiliations:** 1 Department of Medical-Surgical Nursing, College of Nursing, Qassim University, Buraydah, Saudi Arabia; 2 Department of Nursing, Medical City, Qassim University, Buraydah, Saudi Arabia; 3 Bachelor of Science in Nursing, College of Nursing, Qassim University, Buraydah, Saudi Arabia

**Keywords:** migraine disorders, multicenter study, nurses, occupational health, risk perception

## Abstract

**Background:**

Migraine is a common neurological disorder and an emerging occupational health risk among nurses due to demanding workloads, shift work, workplace stress, and prolonged screen exposure. Understanding nurses’ risk perceptions of migraine and its associated occupational factors is essential for workforce health planning and healthcare risk management.

**Aim:**

This study aimed to explore migraine as an occupational health risk among nurses in Saudi Arabia by examining nurses’ risk perceptions and identifying associated demographic, occupational, and lifestyle-related factors across multiple healthcare centers.

**Methods:**

This descriptive cross-sectional multicenter study was conducted and reported in accordance with the Strengthening the Reporting of Observational Studies in Epidemiology (STROBE) guidelines. A convenience sample of 2012 registered nurses in hospitals affiliated with the Qassim Health Cluster. Using a self-administered online survey to assess migraine prevalence, associated occupational risk factors, and related health characteristics. Descriptive and inferential statistical analyses were performed.

**Results:**

Among the 2,012 nurses included in the study, 64.5% were female, and 22.6% were aged 26–34 years. Nurses working in the Emergency Department constituted the largest departmental group (11%). Migraine was reported by 40.9% of participants, with 25.6% attributing symptoms primarily to work-related pressure. Nearly one-quarter (23.3%) reported experiencing migraine for 5 years or longer, while only 31.1% expressed satisfaction with their work environment. Significant relationship was identified between migraine occurrence and working department, daily shift duration, number of patients under care, and availability of assistant nurses. Additionally, 59% of the studied nurses demonstrated unsatisfactory behavioral responses toward migraine coping strategies, indicating potential gaps in effective migraine management among nursing staff.

**Conclusion:**

Migraine is highly prevalent among nurses in Saudi Arabia and is significantly associated with occupational workload, work environment characteristics, and lifestyle-related factors. These findings underscore migraine as an important occupational health risk with implications for workforce well-being, healthcare risk management, and policy development.

## 1 Introduction

Migraine is a primary neurological headache disorder and a major contributor to global disability, affecting nearly one-fifth of the general population worldwide, with women experiencing migraine three to four times more frequently than men (1). It is characterized by recurrent, debilitating attacks often accompanied by nausea, vomiting, and sensitivity to light and sound (2). Beyond episodic pain, migraine has been associated with serious neurological complications, including stroke and hypertension, emphasizing its significance as a public health concern (3). Despite ranking among the related causes of years lived with disability globally, migraine remains underrecognized and inadequately managed in many healthcare systems (2).

From an occupational health perspective, migraine is increasingly recognized as being influenced by workplace exposures such as psychological stress, shift work, workload intensity, sleep disruption, environmental noise, and prolonged screen use (4). Healthcare professionals, particularly nurses, may be especially vulnerable due to the demanding nature of clinical practice, including rotating shifts, high patient acuity, and emotional labor. These occupational factors not only affect nurses’ physical and psychological health but may also influence job satisfaction, workforce retention, burnout, and patient safety outcomes (5).

Globally, migraine prevalence is estimated at approximately 14–15%, contributing significantly to the burden of ill health (6). In Saudi Arabia, migraine prevalence appears relatively high, reaching approximately 37.2% in the general population, with females accounting for a substantial proportion of reported cases (5). Although migraine prevalence has been documented among physicians and the general population in Saudi Arabia, evidence specifically focusing on nurses which the largest segment of the healthcare workforce remains limited (7). This gap is particularly relevant given the occupational exposures nurses face within clinical environments. Individuals with migraine often experience reduced productivity, impaired social functioning, and decreased quality of life (6). Within occupational settings, migraine contributes to absenteeism, and reduced efficiency, while stigma and misconceptions may further contribute to underdiagnosis and undertreatment (3).

Among nurses, headaches and sleep disturbances are frequently reported as indicators of occupational stress. Shift work, particularly rotating and night shifts, disrupts circadian rhythms and has been associated with insomnia, metabolic disturbances, cardiovascular conditions, and increased vulnerability to migraine attacks (8). Additional occupational and lifestyle factors, including excessive workload, irregular sleep patterns, prolonged screen exposure, environmental noise, heat exposure, and stimulant consumption such as caffeine, have also been identified as potential triggers for migraine episodes (9). Although non-pharmacological interventions such as relaxation techniques, cognitive behavioral therapy, and biofeedback have demonstrated effectiveness, their integration into occupational health programs remains limited (10).

Despite the recognized burden of migraine and its impact on workforce productivity and quality of life, it remains insufficiently addressed as an occupational health risk within healthcare systems (6). Nurses in Saudi Arabia operate within a rapidly evolving healthcare system, yet evidence addressing migraine as an occupational health concern in this population remains scarce (11). This lack of evidence limits the ability of healthcare institutions and policymakers to develop targeted occupational health interventions, optimize workforce well-being, and enhance patient safety.

This study is guided by an occupational health framework that conceptualizes migraine as an outcome influenced by both workplace-related and individual determinants. Occupational factors, including clinical department characteristics, workload intensity, shift patterns, patient acuity, and work environment conditions, are considered primary exposures, while individual factors such as age, gender, sleep patterns, stimulant consumption, and lifestyle behaviors may modify migraine risk (7). Nurses’ perceptions of migraine risk are considered an important intermediary factor influencing symptom recognition, coping behaviors, and healthcare-seeking practices (12). Generating evidence in this area is essential for informing occupational health policies, improving nurse well-being, strengthening workforce sustainability, and supporting patient safety.

### 1.1 Aim of the study

This study aimed to explore migraine as an occupational health risk among nurses in Saudi Arabia by examining nurses’ risk perceptions and identifying associated demographic, occupational, and lifestyle-related factors across multiple healthcare centers.

Research questions:

1. What factors are associated with migraine among nurses?
2. What are nurses’ behaviors and occupational risk factors perception related to migraine?
3. Is there a relationship between nurses’ demographic characteristics and migraine risk perception?

## 2 Methods

### 2.1 The study design and setting

This study adopted a descriptive cross-sectional multicenter design and was conducted in major hospitals affiliated with the Qassim Health Cluster in the Qassim region, Saudi Arabia.

### 2.2 Sampling and population

A non-probability convenience sampling approach was utilized to recruit registered nurses working in hospitals affiliated with the Qassim Health Cluster, Qassim region, Saudi Arabia. Eligible participants included practicing registered nurses of both genders, aged between 20 and 60 years, various nationalities and clinical specialties, who were residing and professionally active in study setting during the data collection period and consented to participate in the study. Nurses who were students, interns, not currently engaged in clinical practice, or unwilling to participate were excluded from the study. A total of 2012 eligible nurses were included in the final analysis. A *post hoc* statistical power analysis demonstrated that this sample size provided sufficient power (>80%) to detect small-to-moderate relationship between migraine occurrence and related occupational factors at a significance level of 0.05.

### 2.3 Data collection procedure

Data were collected using a structured, self-administered online questionnaire within a quantitative descriptive cross-sectional study framework. The questionnaire was adapted from previously validated instruments reported in related literature to ensure contextual relevance, clarity, and methodological rigor. Prior to data collection, ethical approval was obtained from the appropriate institutional review authorities, and administrative coordination was established with healthcare facilities affiliated with the Qassim Health Cluster to facilitate study implementation and ensure compliance with institutional and ethical research standards.

The survey was developed and distributed electronically via Microsoft Forms through professional nursing networks, institutional websites, and verified online community platforms targeting nurses working in healthcare settings across the Qassim region, Saudi Arabia. Data collection was conducted over a predefined period from May to September 2025. Completion time for the questionnaire was approximately 3–5 min, which was intended to optimize response rates while minimizing participant burden. Participation was entirely voluntary, responses were anonymous, and no personal identifiable information was collected, thereby ensuring confidentiality and encouraging accurate reporting. Responses were monitored periodically during the data collection phase to ensure completeness, internal consistency, and adherence to the study protocol, supporting the reliability of the collected data. Migraine-related data were assessed based on participants’ self-reported experiences over a defined recent period rather than a single most recent attack, focusing on the occurrence of migraine symptoms, associated triggers, frequency, and treatment use within the occupational context, without aiming to establish clinical diagnosis or evaluate treatment effectiveness. The questionnaire consisted of three main sections.

*Section 1: Demographic characteristics.* This section gathered information on participants’ demographic characteristics, including age, gender, marital status, education level, years of experience, and nationality.

*Section 2: Health-related characteristics and occupational risk factors associated with migraine.* This section assessed health-related factors and occupational risk factors potentially associated with migraine among nurses. Variables included smoking status, stimulant (caffeine) intake, fasting and random blood glucose levels, and blood pressure status. Migraine-related characteristics were also evaluated, including the presence of migraine headache, duration and timing of headache episodes, associated comorbidities, and medications used for symptom management. Additionally, participants reported selected health manifestations commonly linked to migraine and occupational stress, such as headache, allergic rhinitis, tinnitus, dizziness, nausea, vomiting, and blurred vision (4–9).

*Section 3: Nurses’ behavioral experience related to migraine.* This section assessed nurses’ behavioral responses and experiences related to migraine headaches, with a focus on coping strategies. The behavioral scale consisted of seven structured items derived from relevant literature addressing migraine coping and occupational health behaviors, such as appropriate use of prescribed medications, ensuring adequate rest or sleep, minimizing exposure to known triggers (e.g., bright light or noise), stress management through relaxation techniques, maintaining adequate hydration, and seeking medical consultation when symptoms persist. Each item was rated on a three-point frequency scale (usually = 3, sometimes = 2, rarely = 1). Total scores ranged from 7 to 21, with higher scores indicating more appropriate behavioral responses toward migraine management. For interpretation, scores below 60% of the total possible score were classified as unsatisfactory behavior, whereas scores ≥60% were considered satisfactory behavioral responses (2–11).

### 2.4 Pilot study

A pilot study was conducted following instrument development involving approximately 10% of the target nursing population to evaluate clarity, feasibility, and applicability of the questionnaire. Feedback from pilot participants, combined with expert input, informed refinements in wording, structure, and content. Participants included in the pilot study were excluded from the final analysis to minimize potential response bias and ensure data stability.

### 2.5 Validity and reliability

Content validity was established through expert panel review involving specialists in nursing, occupational health, neurology, and research methodology. Panel members independently evaluated each questionnaire item for relevance, clarity, and comprehensiveness using a four-point rating scale. The Content Validity Index (CVI) was calculated at both the item level (I-CVI) and scale level (S-CVI), with values ≥0.78 considered indicative of acceptable content validity. Minor revisions to wording and item sequencing were made based on expert recommendations. Internal consistency reliability was assessed using Cronbach’s alpha coefficient for the overall instrument and relevant subscales. A Cronbach’s alpha value of ≥0.70 was considered acceptable. The findings demonstrated satisfactory internal consistency, indicating that the questionnaire items reliably measured the intended constructs.

*Statistical analysis*: Data were entered, coded, cleaned, and analyzed using IBM SPSS Statistics for Windows, Version 20.0 (IBM Corp., Armonk, NY, United States). Descriptive statistics were used to summarize the study variables. Categorical variables were presented as frequencies and percentages, while continuous variables were summarized using means, standard deviations, and ranges (minimum–maximum values). Comparisons between categorical variables were conducted using the chi-square test (*χ*^2^). When the assumptions for the Chi-square test were not met, the Monte Carlo (MC) exact test was applied. All statistical tests were two-tailed, and statistical significance was considered at *p* ≤ 0.05.

## 3 Results

Table 1 shows that the highest proportion of nurses were aged 35–44 years (27.5%), followed by those aged 26–34 years (22.6%). Females represented the majority (64.5%), followed by males (35.5%). Regarding marital status, married (61.6%) and unmarried nurses (23.9%), with divorced nurses representing (8.2%). In terms of education, most nurses hold a bachelor’s degree (36.4%), followed by diploma (25.0%). The highest proportion had 1–3 years of experience (43.9%), followed by 4–6 years (35.6%). Additionally, the majority were non-Saudi nurses (73.6%), compared with 26.4% Saudi nurses.

**Table 1:** Distribution of the studied nurses according to demographic characteristics (*n* = 2012).

Items	Frequency	
	No.	%
Age		
<25	361	17.9
26–34	455	22.6
35–44	554	27.5
45–54	442	22.0
≥55	200	9.9
Gender		
Male	715	35.5
Female	1,297	64.5
Marital status		
Married	1,239	61.6
Unmarried	481	23.9
Widowed	128	6.4
Divorced	164	8.2
Education level		
Technician	285	14.2
Diploma	503	25.0
Bachelor	732	36.4
Postgraduate	492	24.5
Years of experience		
<1 year	146	7.3
1–3 years	883	43.9
4–5 years	716	35.6
≥6 years	267	13.3
Nationality		
Saudi	531	26.4
Non-Saudi	1,481	73.6

**Figure 1:**
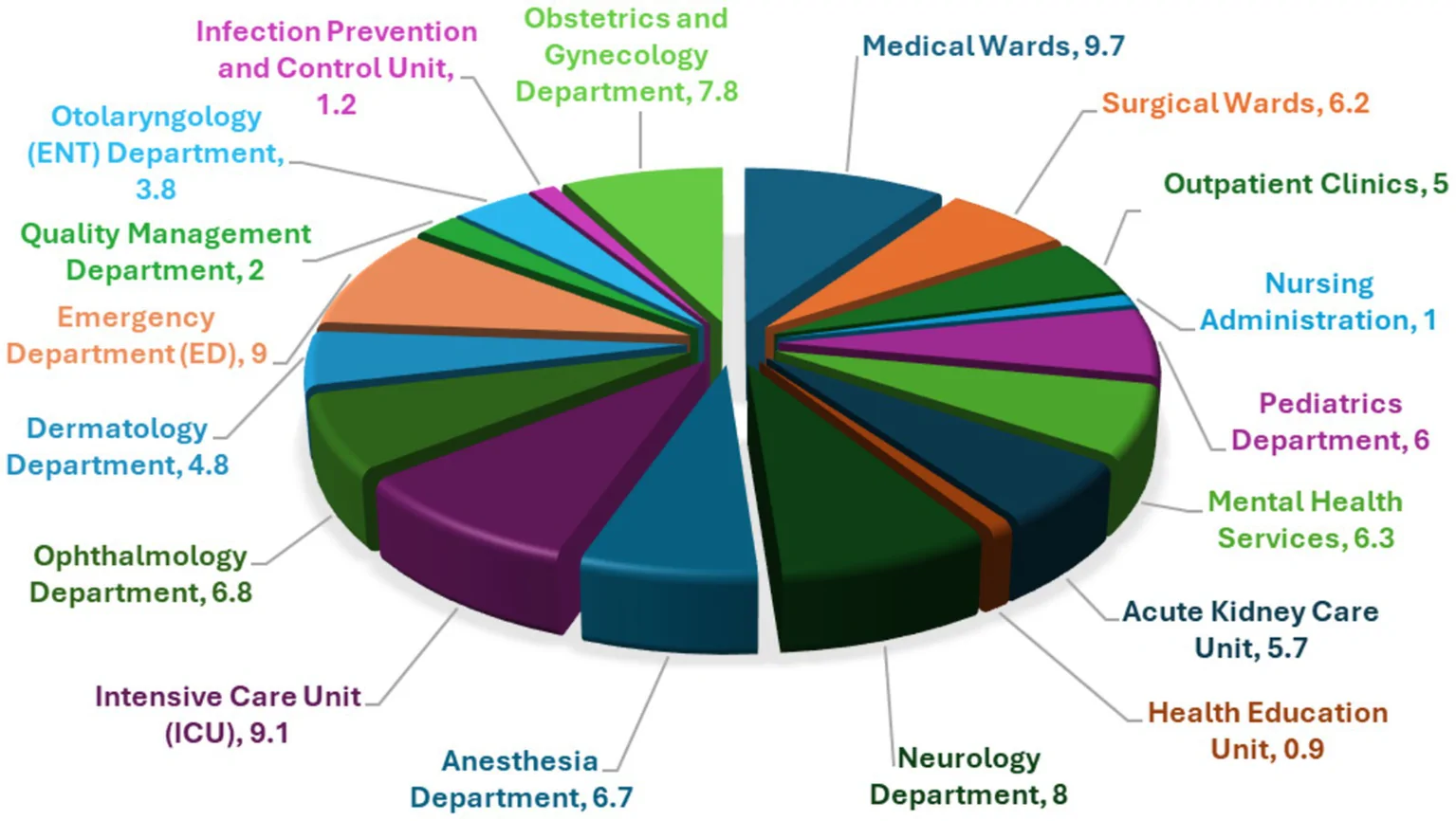
Distribution of the studied nurses by working clinical departments (*n* = 2012).

Figure 1 illustrates that the highest proportions of nurses were working in Medical Wards (9.7%), Intensive Care Units (9.1%), and the Neurology Department (8.0%), followed by the Obstetrics and Gynecology Department (7.8%). Moderate representation was observed in Ophthalmology (6.8%), Anesthesia (6.7%), Mental Health Services (6.3%), and Surgical Wards (6.2%). Lower proportions of nurses were reported in Outpatient Clinics (5.0%), Acute Kidney Care Units (5.7%), Dermatology (4.8%), and Otolaryngology (3.8%), while minimal representation was noted in Quality Management (2.0%), Infection Prevention and Control (1.2%), and Nursing Administration (1.0%).

Table 2 displays that the glycemic status, 25.0% of participants reported an accumulated blood sugar (HbA1c) level of 6.6–7.5%, while 22.1 and 20.2% had levels of 7.6–8.5% and 8.6–9.5%, respectively. More than one-third of nurses (38.5%) reported having high cholesterol, and 42.3% reported high blood pressure. Most participants were non-smokers (60.0%), while 40.0% reported smoking. Regarding migraine management, 38.0% of nurses reported taking medications for migraine. A high proportion of nurses (71.2%) reported consuming stimulant beverages such as coffee, cappuccino, or Nescafé.

**Table 2:** Distribution of the studied nurses according to their health-related characteristics (*n* = 2012).

Items	Frequency	
	No.	%
Accumulated sugar rate (HA1C)		
<6.5%	351	17.4
6.6–7.5%	502	25.0
7.6–8.5%	444	22.1
8.6–9.5%	406	20.2
≥9.6%	309	15.4
Do you have high cholesterol?		
Yes	775	38.5
No	739	36.7
Do you have high blood pressure?		
Yes	851	42.3
No	1,161	57.7
Do you smoke?		
Yes	805	40.0
No	1,207	60.0
Do you take medications for migraine?		
Yes	774	38.0
No	1,238	62.0
Do you drink stimulants such as coffee, cappuccino, or Nescafe?		
Yes	1,432	71.2
No	317	15.8
Sometimes	263	13.1

**Figure 2:**
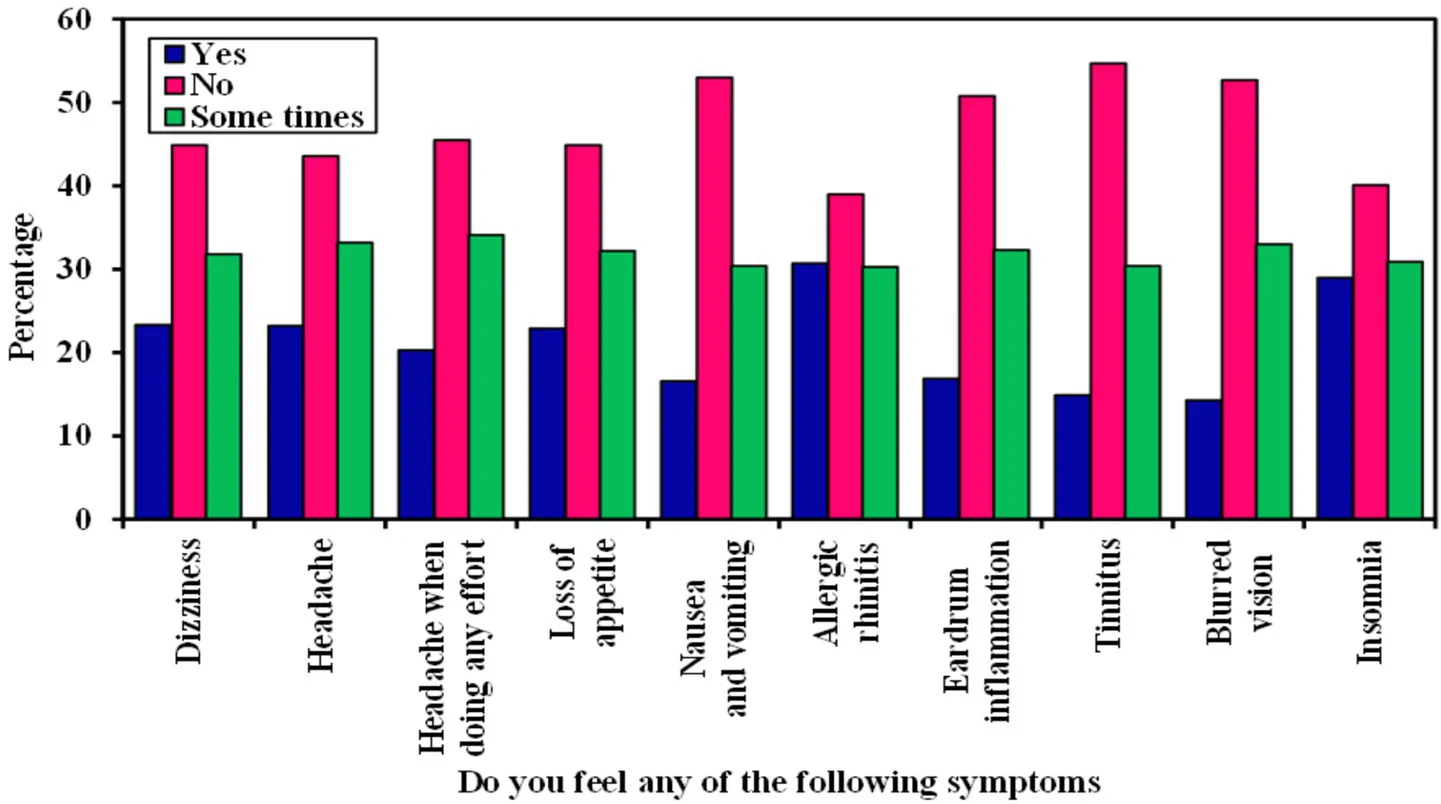
Distribution of the studied nurses according to their feeling or complaining symptoms (*n* = 2012).

Figure 2 illustrates the distribution of nurses according to self-reported symptoms and complaints. The most frequently reported conditions were allergic rhinitis (33%) and insomnia (32%), followed by dizziness, headache, and loss of appetite (22% each).

Table 3 presents that most nurses worked 8 h per day (37.5%), followed by 16-h shifts (30.0%). The evening shift was the most preferred work period (38.1%). Nearly one-third of nurses (31.5%) reported being responsible for five or more patients per shift. Regarding migraine status, 40.9% of nurses reported experiencing migraine headache, while 36.4% reported experiencing migraine occasionally. Work-related pressure was the most reported trigger (25.6%). Approximately 23.3% of nurses reported suffering from migraine for 5 years or longer, and only 31.1% were satisfied with their work environment. Daily computer or laptop use of 3 hours or more was reported by most participants (33.3%).

**Table 3:** Distribution of the studied nurses according to occupational risk factors (*n* = 2012).

Items	Frequency	
	No.	%
Workings hours per day		
4 h	440	21.9
8 h	755	37.5
16 h	604	30.0
24 h	213	10.6
Favorite time to work		
Morning	626	31.1
Night	619	30.8
Evening	769	38.1
Number of patients responsible for		
One patient	377	18.7
Tow	475	23.6
From 2 to 4	527	26.2
5 patients or more	633	31.5
Do you suffer from migraine?		
Yes	822	40.9
No	457	22.7
Sometimes	733	36.4
If the answer is yes. What time do you feel like a migraine?		
Morning	265	13.2
Evening	258	12.8
Afternoon	301	15.0
Exposure to work pressure	516	25.6
During menstruation period	215	10.7
I do not feel a migraine	457	22.7
How long have you been suffering from migraine?		
One year	382	19.0
Two years	326	16.2
From 2 to 4 years	398	19.8
5 years and above	469	23.3
I do not feel headaches	437	21.7
How satisfied are you with your work and work environment?		
Very satisfied	263	13.1
Satisfied	625	31.1
Neutral	393	19.5
Not satisfied	413	20.5
Very dissatisfied	318	15.8
How often do you work on the computer or laptop daily?		
3 h	669	33.3
4–7 h	583	29.0
7–10 h	280	13.9
More than 10 h	368	18.3
I do not work on the computer	112	5.6

Table 4 presents that there is a statistically significant association between several demographic characteristics and both nurses’ behavior toward migraine and occupational risk factors. Significant relationships were observed with gender (behavior: *χ*^2^ = 5.89, *p* = 0.01; occupational risk factors: *χ*^2^ = 4.92, *p* = 0.05), clinical departments (behavior: *χ*^2^ = 62.61, *p* = 0.001; occupational risk factors: *χ*^2^ = 12.01, *p* = 0.01), years of experience (behavior: *χ*^2^ = 14.62, *p* = 0.002; occupational risk factors: *χ*^2^ = 11.11, *p* = 0.01), and nationality (behavior: *χ*^2^ = 17.63, *p* = 0.001; occupational risk factors: *χ*^2^ = 13.87, *p* = 0.01). However, age, marital status, and educational level did not show statistically significant relationship with nurses’ behavior toward migraine or occupational risk factors.

**Table 4:** Relationship between the studied nurses’ demographic characteristics with behaviors toward migraine and occupational risk factors perception (*n* = 2012).

Items	Frequency		Nurse’s behavior towards migraine		Occupational risk factors	
	No.	%	*χ* ^2^	*p*	*χ* ^2^	*p*
Age						
<25	361	17.9	3.07	0.54	16.47	0.82
26–34	455	22.6				
35–44	554	27.5				
45–54	442	22.0				
>55	200	9.9				
Gender						
Male	715	35.5	5.89*	0.01*	4.92*	0.05*
Female	1,297	64.5				
Marital status						
Married	1,239	61.6	3.29	0.66	7.20	0.51
Unmarried	481	23.9				
Widowed	128	6.4				
Divorced	164	8.2				
Departments			62.61*	0.001*	12.01*	0.01*
Education level						
Technician	285	14.2	7.11	0.068	8.26	0.086
Diploma	503	25.0				
Bachelor	732	36.4				
Postgraduate	492	24.5				
Years of experience						
<1 year	146	7.3	14.61*	0.002*	11.11*	0.01*
1–3 years	883	43.9				
4–5 years	716	35.6				
≥6 years	267	13.3				
Nationality						
Saudi	531	26.4	17.63*	0.001*	13.87*	0.01*
Non-Saudi	1,481	73.6				

*χ*^2^, chi-square test; *, statistically significant at *p* ≤ 0.05.

Table 5 exhibits significant relationship between several demographic characteristics and suffering from migraine among nurses. Age demonstrated a statistically significant relationship (*χ*^2^ = 15.74, MC *p* = 0.015), indicating variation in migraine occurrence across different age groups. Marital status (*χ*^2^ = 99.06, *p* < 0.001) and clinical department (*χ*^2^ = 67.35, *p* = 0.001) were also significantly associated with migraine, suggesting the influence of social and occupational factors. Additionally, years of experience (*χ*^2^ = 18.29, MC *p* = 0.006) and nationality (*χ*^2^ = 31.90, *p* < 0.001) showed significant relationships with migraine occurrence. However, gender (*χ*^2^ = 1.13, *p* = 0.568) and educational level (*χ*^2^ = 6.15, *p* = 0.407) were not significantly associated with suffering from migraine among the studied nurses.

**Table 5:** Relationship between the studied nurses’ demographic characteristics and suffering from migraine (*n* = 2012).

Items	Suffering from migraine						*χ* ^2^	*p*
	Yes (*n* = 822)		No (*n* = 457)		Sometimes (*n* = 733)			
	No.	%	No.	%	No.	%		
Age								
<25	184	22.4	73	16.0	104	14.2	15.74*	MC *p* = 0.015*
26–34	171	20.8	114	24.9	170	23.2		
35–44	214	26.0	128	28.0	212	28.9		
45–54	172	20.9	106	23.2	164	22.4		
>55	81	9.9	36	7.9	83	11.3		
Gender								
Male	282	34.3	170	37.2	263	35.9	1.132	0.568
Female	540	65.7	287	62.8	470	64.1		
Marital status							99.06*	<0.001*
Married	402	32.4	459	37.0	378	30.5		
Unmarried	123	25.6	156	32.4	202	42.0		
Widowed	31	24.2	35	27.3	62	48.4		
Divorced	36	22.0	54	32.9	74	451		
Department							67.35*	0.001*
Education level								
Technician	87	10.6	36	7.9	62	8.5	6.148	0.407
Diploma	223	27.1	131	28.7	229	31.2		
Bachelor	209	25.4	113	24.7	181	24.7		
Postgraduate	303	36.9	177	38.7	261	35.6		
Years of experience								
<1 year	46	31.5	52	35.6	48	32.9	18.29*	MCp = 0.006*
1–3 years	282	31.9	398	45.1	203	23.0		
4–5 years	267	37.3	323	45.1	126	17.6		
≥6 years	56	21.0	135	50.6	76	28.5		
Nationality								
Saudi	160	30.1	238	44.8	133	25.0	31.90*	<0.001*
Non-Saudi	396	26.7	439	29.6	646	43.6		

*χ*^2^, chi-square test; MC, Monte Carlo test; *, significant at *p* ≤ 0.05.

Table 6 demonstrates that there is a statistically significant relationship were observed between stimulant intake and several symptoms, including dizziness (*χ*^2^ = 48.39, *p* < 0.001), headache (*χ*^2^ = 16.66, *p* = 0.002), nausea and vomiting (*χ*^2^ = 14.99, *p* = 0.005), allergic rhinitis (*χ*^2^ = 16.71, *p* = 0.002), otitis media (*χ*^2^ = 16.35, *p* = 0.003), tinnitus (*χ*^2^ = 59.54, p < 0.001), and blurred vision (*χ*^2^ = 45.10, p < 0.001). No statistically significant relationship were found between stimulant consumption and loss of appetite (*p* = 0.104) or insomnia (*p* = 0.370).

**Table 6:** Relation between the studied nurse’s complaints or discomfort problems and drink stimulants (*n* = 2012).

Complaints or discomfort problems	Drink stimulant						*χ* ^2^	*p*
	Yes (*n* = 1,432)		No (*n* = 317)		Sometimes (*n* = 263)			
	No.	%	No.	%	No.	%		
Dizziness								
Yes	367	25.6	54	17.0	48	18.3	48.39*	<0.001*
No	665	46.4	148	46.7	90	34.2		
Sometimes	400	27.9	115	36.3	125	47.5		
Headache								
Yes	317	22.1	94	29.7	55	20.9	16.66*	0.002*
No	609	42.5	139	43.8	130	49.4		
Sometimes	506	35.3	84	26.5	78	29.7		
Loss appetite								
Yes	329	23.0	76	24.0	56	21.3	7.67	0.104
No	619	43.2	151	47.6	133	50.6		
Sometimes	484	33.8	90	28.4	74	28.1		
Nausea and vomiting								
Yes	259	18.1	42	13.2	32	12.2	14.99*	0.005*
No	768	53.6	163	51.4	136	51.7		
Sometimes	405	28.3	112	35.3	95	36.1		
Allergic rhinitis								
Yes	430	30.0	94	29.7	93	35.4	16.71*	0.002*
No	594	41.5	105	33.1	86	32.7		
Sometimes	408	28.5	118	37.2	84	31.9		
Otitis media								
Yes	236	16.5	57	18.0	48	18.3	16.35*	0.003*
No	763	53.3	132	41.6	127	48.3		
Sometimes	433	30.2	128	40.4	88	33.5		
Tinnitus								
Yes	172	12.0	75	23.7	52	19.8	59.54*	<0.001*
No	852	59.5	122	38.5	127	48.3		
Sometimes	408	28.5	120	37.9	84	31.9		
Blurred vision								
Yes	158	11.0	70	22.1	60	22.8	45.10*	<0.001*
No	784	54.7	145	45.7	131	49.8		
Sometimes	490	34.2	102	32.2	72	27.4		
Insomnia								
Yes	411	28.7	87	27.4	85	32.3	4.27	0.370
No	565	39.5	140	44.2	102	38.8		
Sometimes	456	31.8	90	28.4	76	28.9		

*χ*^2^, chi square test; *, significant at *p* ≤ 0.05.

**Figure 3:**
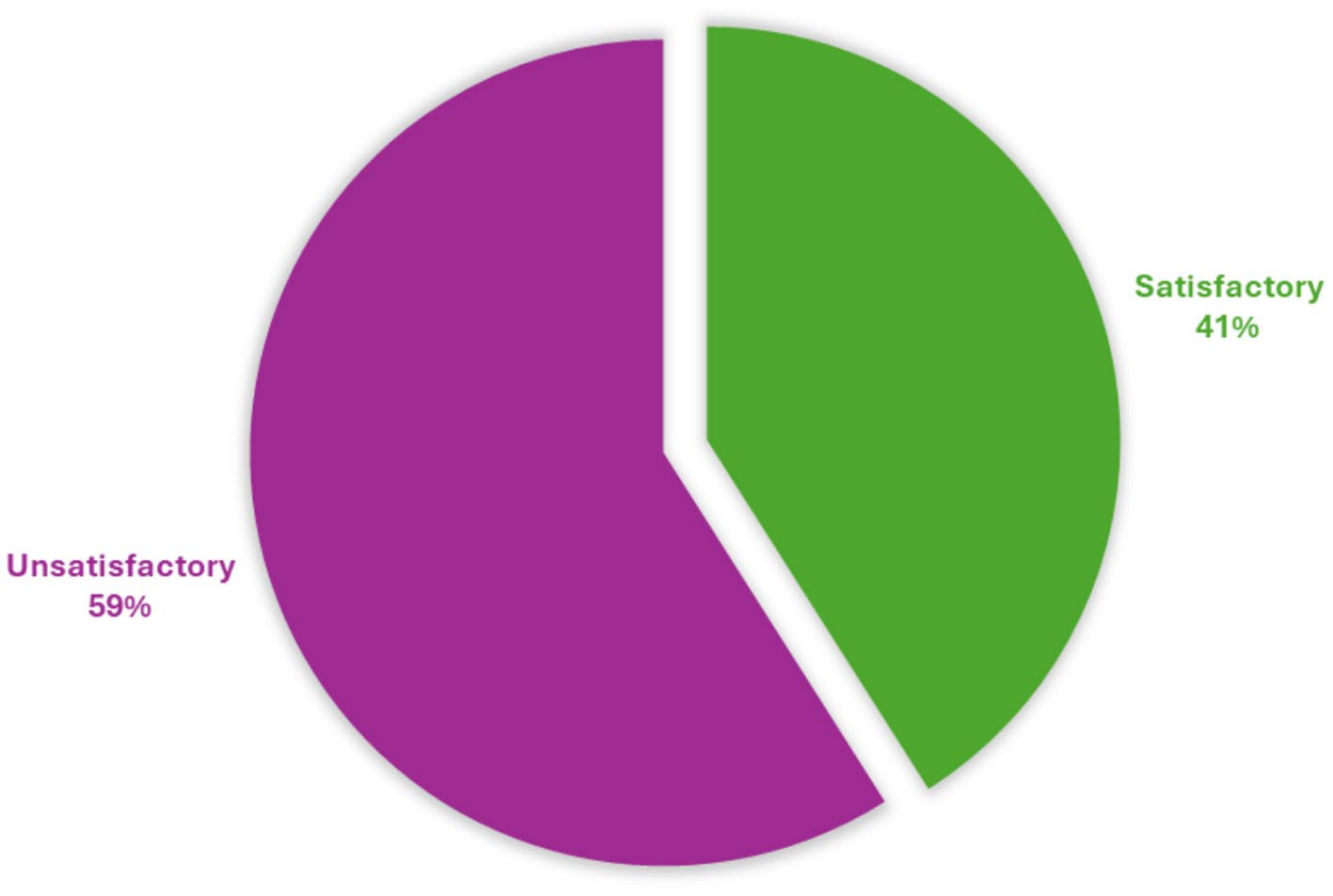
Level of the studied nurses’ behavioral experience related to migraine (*n* = 2012).

Figure 3 shows that approximately 41% of nurses demonstrated satisfactory behavioral responses toward migraine coping strategies, whereas about 59% exhibited unsatisfactory behavioral response.

## 4 Discussion

Regarding the demographic characteristics of the studied nurses, the predominance of nurses in the 35–44-year age group followed by those aged 26–34 years reflects a largely mid-career workforce, which is consistent with nursing workforce trends reported in previous studies. The higher proportion of female nurses aligns with global nursing demographics and may have implications for migraine risk, as migraine is generally more prevalent among women (3–12). The distribution across marital status categories suggests diverse social backgrounds that may influence stress exposure and health outcomes. In terms of education, the predominance of bachelor-prepared nurses reflects the increasing professionalization of nursing practice. Additionally, the large proportion of nurses with 1–3 years of experience may indicate early-career exposure to occupational stressors. The predominance of non-Saudi nurses is consistent with workforce patterns in Saudi healthcare systems in similar studies, where expatriate nurses form a substantial part of the clinical workforce (6–13).

Concerning the health-related characteristics of the studied nurses (Table 2), a considerable proportion reported elevated HbA1c levels, with notable percentages within moderate glycemic ranges, while more than one-third reported high cholesterol and a substantial proportion reported hypertension. The majority of nurses were non-smokers, although a significant proportion reported smoking. Additionally, a notable percentage reported using medications for migraine, and a high proportion reported consuming stimulant beverages such as caffeinated drinks. Similar studies have indicated that metabolic risk factors, caffeine intake, and occupational stress may contribute to migraine occurrence among healthcare workers (14). However, other studies have reported inconsistent relationship between metabolic indicators and migraine, suggesting that lifestyle, sleep quality, and psychological stress may play a more dominant role (9–15).

About nurses’ self-reported symptoms and complaints (Figure 2), the most frequently reported conditions were allergic rhinitis and insomnia, followed by dizziness, headache, and loss of appetite. Similar studies have shown that sleep disturbances and allergic conditions are commonly associated with migraine and occupational stress among healthcare workers (12–16). Insomnia, in particular, has been identified as a significant trigger for migraine due to circadian rhythm disruption and workload demands. However, some studies have reported inconsistent relationship between these symptoms and migraine, suggesting that individual susceptibility, environmental exposure, and psychological factors may also influence symptom occurrence (8–17).

Concerning occupational risk factors (Table 3), the findings indicate that most nurses worked standard 8-h shifts, while a considerable proportion reported extended 16-h shifts, with evening duty being the most common work period. A notable proportion of nurses were responsible for multiple patients per shift, reflecting substantial workload demands. Additionally, a high percentage reported experiencing migraine or occasional migraine, with work-related pressure identified as the most frequent trigger. Similar studies have shown that shift work, heavy workload, and occupational stress are important contributors to migraine among healthcare professionals (18, 19). However, some research suggests inconsistent relationship between shift patterns and migraine, highlighting the potential influence of individual susceptibility, coping mechanisms, and lifestyle factors (20, 21). These findings emphasize the multifactorial occupational influences on migraine among nurses.

Interpreting the relationship between demographic characteristics, nurses’ behavior toward migraine (Table 4), and occupational risk factors, the findings showed significant relationship with gender, clinical department, years of experience, and nationality. Similar studies have reported that workplace environment, professional experience, and gender differences may influence health awareness, coping behaviors, and exposure to occupational stressors among nurses (22, 23). In contrast, age, marital status, and educational level were not significantly associated, which aligns with other studies suggesting that occupational conditions may play a greater role than demographic factors in migraine-related outcomes (4–24). These findings emphasize the importance of workplace context and professional experience in understanding migraine risk and behavioral responses among nursing professionals.

Regarding the relationship between demographic characteristics and suffering from migraine (Table 5), the findings showed significant relationship with age, marital status, clinical department, years of experience, and nationality, suggesting that both social and occupational factors may influence migraine occurrence among nurses. Similar studies have reported that work environment, professional experience, and occupational stress exposure are important contributors to migraine prevalence in healthcare workers (5–25). However, the absence of significant relationship with gender and educational level aligns with some research indicating that migraine risk may be more strongly related to workplace conditions and lifestyle factors rather than demographic characteristics alone (26). These findings highlight the multifactorial nature of migraine among nursing professionals.

Concerning the relationship between stimulant intake and nurses’ complaints or discomfort symptoms (Table 6), the findings showed significant relationship with several symptoms, including dizziness, headache, nausea and vomiting, allergic rhinitis, otitis media, tinnitus, and blurred vision, suggesting a potential link between stimulant consumption and these health complaints. Similar studies have reported that excessive caffeine intake may trigger headaches, dizziness, and sensory symptoms, particularly among healthcare workers exposed to occupational stress and irregular work schedules (27, 28). However, no significant relationship was observed with loss of appetite or insomnia, which is consistent with other related studies indicating that caffeine effects may vary depending on individual tolerance and consumption patterns (29, 30). These findings highlight the need to consider stimulant intake as a potential contributing factor to migraine-related symptoms among nurses.

Interpreting nurses’ behavioral experiences related to migraine coping strategies, the findings indicate that less than half of the nurses demonstrated satisfactory behavioral responses, while the majority showed unsatisfactory coping practices. This may reflect gaps in awareness, occupational stress, workload demands, or limited access to structured occupational health support. Similar studies have reported that healthcare professionals often adopt suboptimal coping strategies due to demanding work environments, shift work, and fatigue, which may negatively influence migraine management (11–20). Inadequate coping behaviors have been associated with increased migraine frequency, reduced productivity, and impaired quality of life. However, some studies suggest variability in coping behaviors depending on individual health literacy, workplace support, and organizational culture (8–26). From the authors’ perspective, the predominance of unsatisfactory behavioral responses highlights the need for targeted educational programs, stress management interventions, and workplace health promotion initiatives.

*The study limitations*. The authors acknowledge several limitations of this study. First, the cross-sectional design limits the ability to establish causal relationships between migraine and the associated occupational factors. Second, the use of convenience sampling may restrict the generalizability of the findings to the broader nursing population. In addition, reliance on self-reported data may introduce recall and reporting bias. Furthermore, migraine identification was based on participants’ self-reports rather than clinical diagnosis or differentiation of specific headache types using standardized criteria, which may have resulted in potential underestimation or overestimation of the true prevalence. Future longitudinal and interventional studies are encouraged to apply multivariate modeling approaches to better identify independent risk factors for migraine among nurses and to inform strategies aimed at improving workforce health and well-being.

## 5 Conclusion and recommendations

The findings of this multicenter study indicate that migraine is a common health concern among nurses in Saudi Arabia and is associated with several demographic and occupational factors, including clinical department, workload demands, shift duration, patient load, and workplace environment. The relatively high prevalence of migraine, together with the notable proportion of nurses reporting unsatisfactory coping behaviors, highlights migraine as a relevant occupational health issue that may influence nurses’ well-being, job performance, and healthcare service quality. These findings reinforce the importance of recognizing migraine not only as an individual health problem but also as a workplace-related concern within healthcare systems.

Accordingly, integrating migraine awareness, prevention, and management strategies into occupational health programs is recommended. Healthcare institutions should consider optimizing staffing levels, improving shift scheduling practices, promoting healthy sleep and stress management strategies, and providing educational programs aimed at enhancing nurses’ coping behaviors and early symptom recognition. Supportive work environments and access to occupational health resources may help mitigate migraine burden and improve workforce sustainability. Future research should employ longitudinal and interventional designs to better understand causal pathways, evaluate targeted occupational health interventions, and inform evidence-based policies aimed at improving nurses’ health outcomes and patient care quality.

### 5.1 Practice implications

Migraine among nurses represents an important occupational health concern with potential implications for workforce sustainability, productivity, and patient safety. The associations observed between migraine occurrence and factors such as workload intensity, shift duration, staffing levels, clinical department, and lifestyle characteristics suggest that migraine should be considered not only an individual health issue but also a workplace-related concern requiring organizational attention.

In alignment with Saudi Vision 2030 priorities for healthcare quality and workforce well-being, healthcare institutions may benefit from integrating migraine awareness, prevention, and management strategies into occupational health programs. Practical measures could include optimizing nurse workloads, improving staffing ratios, refining shift scheduling practices, particularly minimizing prolonged or rotating shifts where feasible and promoting healthy sleep, stress management, and responsible stimulant consumption. Educational initiatives focused on early symptom recognition and effective coping strategies may further support nurse well-being. Adopting proactive occupational health approaches may help reduce migraine-related absenteeism and presenteeism, enhance nurse retention, and contribute to improved.

## Data Availability

The original contributions presented in the study are included in the article/supplementary material, further inquiries can be directed to the corresponding author.
